# Comparative Genomics Identifies the Evolutionarily Conserved Gene *TPM3* as a Target of eca-miR-1 Involved in the Skeletal Muscle Development of Donkeys

**DOI:** 10.3390/ijms242015440

**Published:** 2023-10-22

**Authors:** Ge Yang, Minhao Sun, Zhaofei Wang, Qiaoyan Hu, Jiajun Guo, Jie Yu, Chuzhao Lei, Ruihua Dang

**Affiliations:** Key Laboratory of Animal Genetics, Breeding and Reproduction of Shaanxi Province, College of Animal Science and Technology, Northwest A&F University, Yangling, Xianyang 712100, China; geyang0125@nwafu.edu.cn (G.Y.); sunminhao@nwafu.edu.cn (M.S.); wangzhaofei2016@nwafu.edu.cn (Z.W.); hqy0124@nwsuaf.edu.cn (Q.H.); 1219076829@nwafu.edu.cn (J.G.); 1138963341@dongeejiao.com (J.Y.); leichuzhao1118@nwafu.edu.cn (C.L.)

**Keywords:** *Equus*, comparative genomics, skeletal muscle development, *TPM3*, eca-miRNA-1

## Abstract

Species within the genus *Equus* are valued for their draft ability. Skeletal muscle forms the foundation of the draft ability of *Equus* species; however, skeletal muscle development-related conserved genes and their target miRNAs are rarely reported for *Equus*. In this study, a comparative genomics analysis was performed among five species (horse, donkey, zebra, cattle, and goat), and the results showed that a total of 15,262 (47.43%) genes formed the core gene set of the five species. Only nine chromosomes (Chr01, Chr02, Chr03, Chr06, Chr10, Chr18, Chr22, Chr27, Chr29, and Chr30) exhibited a good collinearity relationship among *Equus* species. The micro-synteny analysis results showed that *TPM3* was evolutionarily conserved in chromosome 1 in *Equus*. Furthermore, donkeys were used as the model species for *Equus* to investigate the genetic role of *TPM3* in muscle development. Interestingly, the results of comparative transcriptomics showed that the *TPM3* gene was differentially expressed in donkey skeletal muscle S1 (2 months old) and S2 (24 months old), as verified via RT-PCR. Dual-luciferase test analysis showed that the *TPM3* gene was targeted by differentially expressed miRNA (eca-miR-1). Furthermore, a total of 17 *TPM3* gene family members were identified in the whole genome of donkey, and a heatmap analysis showed that *EaTPM3-5* was a key member of the *TPM3* gene family, which is involved in skeletal muscle development. In conclusion, the *TPM3* gene was conserved in *Equus*, and *EaTPM3-5* was targeted by eca-miR-1, which is involved in skeletal muscle development in donkeys.

## 1. Introduction

The *Equus* genus comprises donkeys, horses, and zebras [1]. The chromosomal-level genomes of horses, donkeys, and zebras allow for the investigation of the karyotype or chromosomal evolution, which is important for understanding *Equus* genome organization and chromosomal architecture [2,3]. Donkey reference genome research concerns investigating the genetic basis of coat color and population genomics analyses [1]. The quality of donkey and zebra genomes has significantly improved; however, they lack a comparative study [2,3,4]. Comparative genomics analysis of mammals can identify genes with conserved functions, especially among Equine animals, though few studies exist [5,6]. Previous studies have used collinearity analysis to identify the role of evolutionarily conserved genes [7,8]. Skeletal muscle in the *Equus* genus plays an important role as a dynamic tissue in the body, and exercise ability is very important for donkeys and horses, which drives their important economic value [9,10]. An analysis of the fatty acid composition of donkey intramuscular fat showed that the content of polyunsaturated fatty acids (PUFAs) in both longissimus dorsi and biceps femoris muscles at 25.16 g/100 g and 24.97 g/100 g total fatty acids, respectively [9]. Therefore, it is necessary to use comparative genomics to identify conserved genes for muscle development in *Equus* species.

Many studies have utilized transcriptomic analyses to identify key candidate enzyme genes and miRNAs for muscle development [11,12,13,14]. In pigs, a total of 99 differentially expressed genes and 15 differentially expressed miRNAs (DE-miRNAs) were identified [11]. Another study also reported a total of 85 genes and 18 miRNAs related to muscle growth [12]. In chickens, a previous study identified that 337 miRNAs are differentially expressed during muscle development [13]. Moreover, the gga-miR-499-5p/SOX6 and gga-miR-196-5p/CALM1 networks are involved in the determination of muscle fiber types [14]. Therefore, it is necessary to identify genes and miRNAs involved in muscle development by comparing transcriptomes.

A-tropomyosin-3 (*TPM3*) is an actin-binding protein that plays a crucial role in the regulation of muscle development [15,16]. In myostatin-edited Meishan pigs, *TPM3* participates in the regulation of muscle growth and development. In rabbits, *TPM3* exhibits stronger protein signals in fetal hearts and adult skeletal muscle compared to adult hearts [15]. Slow skeletal muscles are associated with mutations in the *TPM3* gene [17]. In donkeys, *TPM3* is differentially expressed in different skeletal muscle types [18]. Studies have shown that the *TPM3* gene is associated with muscle development in many animals [15,16,17,18]. Therefore, in order to identify the functional genes related to muscle development in the *Equus* genus, it is necessary to study *TPM3*. MiRNAs can also bind to other recognition positions of target genes, such as 5′UTR [19], promoters [20], and open reading frames [21,22,23]. MiRNAs play roles in both the cytoplasm and nucleus of cells to regulate the expression of the corresponding genes [24,25]. An increasing number of miRNAs have been found to play important regulatory roles in skeletal muscle development. MiR-206 and miR-208a play an important role in knocking out the Dicer enzyme, which causes muscle underdevelopment or death in newborn mice [26]; studies have also found that a large number of highly expressed miRNAs, such as miR-206 and miR-208a, are excavated in cardiac or skeletal muscle [27]. MiR-7 plays a regulatory role in muscle disease [28], and miR-434-3p targets the eIF5A1 gene to promote skeletal muscle apoptosis [29]. MiR-125b targets insulin-like growth factor II (IGF2) to inhibit myoblast differentiation and skeletal muscle regeneration [30], and it was also found that the upregulation of miR-199a-3p promotes the transformation of muscle fiber types [31]. MiR-29 targets Akt3 to promote myoblast differentiation and inhibit myoblast proliferation [32], miR-638 inhibits muscle cell glycolysis by targeting the lactate dehydrogenase (LDHA) gene [33], miR-3646 promotes the proliferation and migration of vascular smooth muscle cells by directly targeting the rho-related GTP-binding (RHOH) gene [34], miR-210 inhibits smooth muscle cell apoptosis by targeting the myocyte enhancer factor 2 (MEF2C) gene [35], and miR-885 promotes the proliferation and inhibition of myoblast differentiation by targeting the myogenic differentiation 1 (MyoD1) gene [36]. These studies suggest that miRNAs play an important role in skeletal muscle regeneration, as well as myoblast differentiation and proliferation.

A previous study using lncRNA–miRNA–mRNA interaction network analysis showed that there were three important candidate lncRNAs (MSTRG.9787.1, MSTRG.3144.1, and MSTRG.9886.1) and candidate gene *Alpha-actinin 1* (*ACTN1*) involved in the skeletal muscle in donkeys [18]. Therefore, it is necessary to identify more miRNAs and genes involved in the muscle development of the *Equus* genus. In this study, we performed comparative genomics to both investigate chromosomal evolution and identify the role of evolutionarily conserved genes in the *Equus* genome, especially in muscle development. To investigate the mechanism of muscle development in *Equus*, this study employs a comparative transcriptomics analysis using skeletal muscle transcriptome data from donkeys of different ages. We characterized the expression of different genes and miRNAs and their networks using transcriptomics, RT-PCR, and the dual-luciferase reporter assay. Furthermore, the *TPM3* gene family number was identified as playing a critical role in *TPM3* gene-related muscle development in donkeys.

## 2. Results

### 2.1. Comparative Genomics of Equine Genomes and Outgroup (Goat and Cattle)

**Figure 1:**
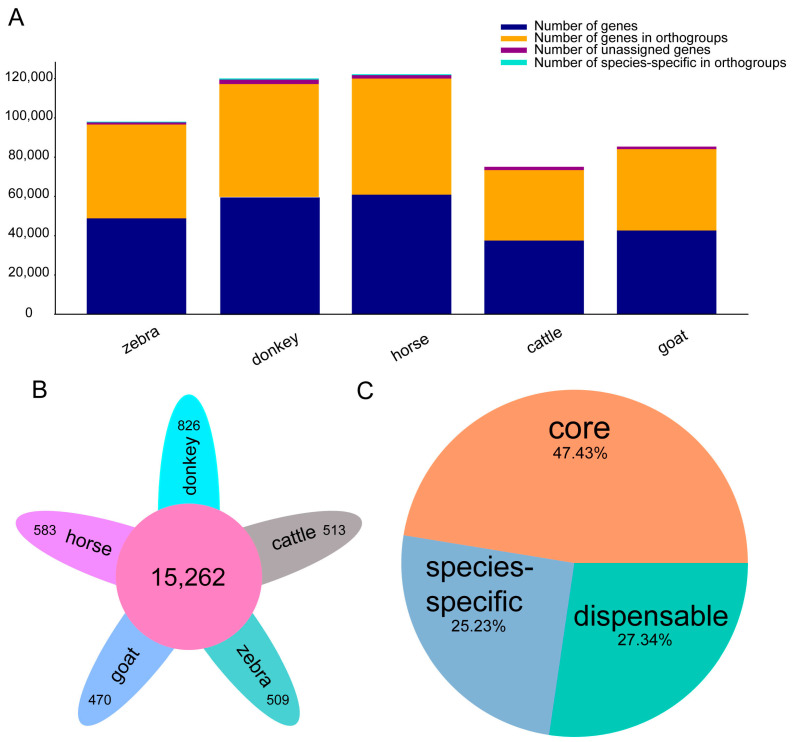
*Equus* genome evolution. (**A**) The percentage of different gene types among the five species. (**B**) The homologous genes in donkeys, cattle, zebras, goats, and horses. (**C**) The core and species-specific genes. Orange, light green, and blue are the core, dispensable, and species-specific gene families, respectively, among the five species.

Comparative genomics of the five species (*Equus caballus* (horse), *Bos taurus* (cattle), *Equus quagga* (zebra), *Equus asinus* (donkey), and *Capra hircus* (goat)) was performed based on protein-coding genes. In the analysis of the 49,384 gene families among them, the five species contain 23,805–25,042 gene families in each genome (Figure 1A), 15,262 (47.43%) of which are shared among all the species, probably representing the core gene set of the five species (Figure 1B). In addition, a total of 20,086 (27.33%) dispensable and 17,101 (25.23%) species-specific gene families were also identified from these species (Figure 1C).

### 2.2. The Karyotype Evolution of Equine Genomes

**Figure 2:**
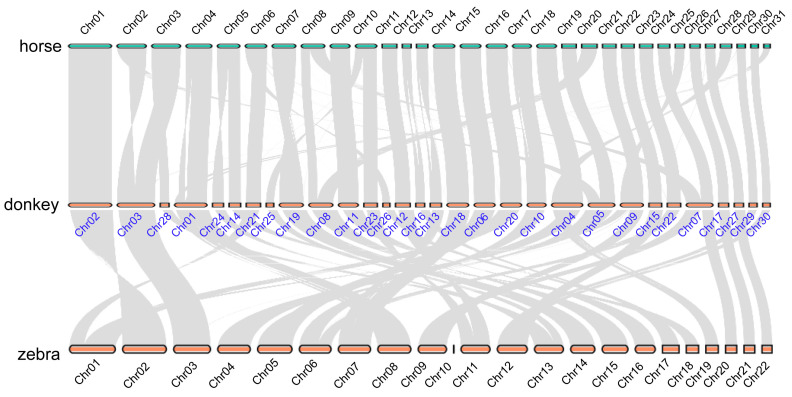
The chromosome evolution events among the *Equus* species. Genome collinearity among horse, donkey, and zebra.

We reconstructed the evolutionary history of chromosomal changes among the three *Equus* species, which included horse, zebra, and donkey (Figure 2). A number of rearrangements were found in the three *Equus* species, indicating that multiple chromosomal fusion or fission events occurred (Figure 2). The frequency of chromosomal rearrangements varied among chromosomes, with some chromosomes experiencing repeated and independent rearrangements. We found that, in donkeys, nine chromosomes (Chr01, Chr02, Chr03, Chr06, Chr10, Chr18, Chr22, Chr27, Chr29, and Chr30) showed a good collinearity relationship among equine genomes. Besides these chromosomes, frequent chromosomal changes were shown among the three *Equus* species (Figure 2).

### 2.3. TPM3 Evolutionarily Conserved in Equus and Differentially Expressed in S1 and S2 Muscle

**Figure 3:**
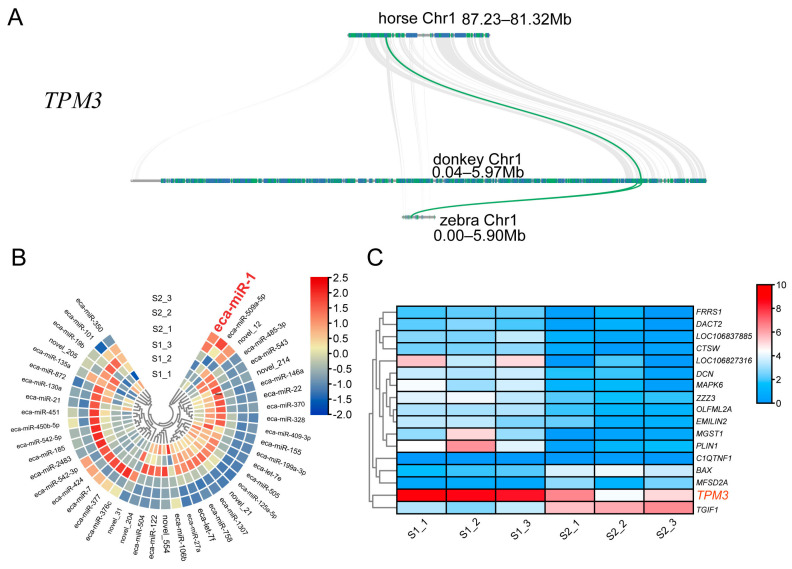
Comparative genomics identified evolutionarily conserved gene *TPM3*. (**A**) *TPM3* gene collinearity among horses, donkeys, and zebras. (**B**) Differentially expressed miRNAs in S1 and S2 transcriptome data. (**C**) The differentially expressed genes in S1 and S2 transcriptome data. S1 indicates muscle from a 2−month-old donkey; S2 indicates muscle from a 24−month-old donkey.

While various chromosomes underwent significant changes in the three *Equus* species, the genes related to skeletal muscle development remained evolutionarily conserved. In our study, the collinearity analysis result showed that *TPM3*, which plays an important role in muscle development, was evolutionarily conserved in the five animal species (Figure 3A,B). Based on our transcriptome data of S1 and S2, we identified 45 miRNAs that were differentially expressed between these two transcriptomes (Figure 3C). Among the differentially expressed miRNAs, three miRNAs were highly expressed in S2-staged muscle compared to S1-stage muscle, which included eca-miR-1, eca-miR-509a-5p, and novel_12 (Figure 3C). Additionally, a total of 17 genes showed differential expression between the two transcriptomes. Among them, the *TPM3* gene showed higher expression in S1 muscle than S2 muscle, indicating that the eca-miR-1 and *TPM3* genes play a critical role in muscle development (Figure 3).

### 2.4. Eca-miR-1 Targeting the TPM3 Gene

**Figure 4:**
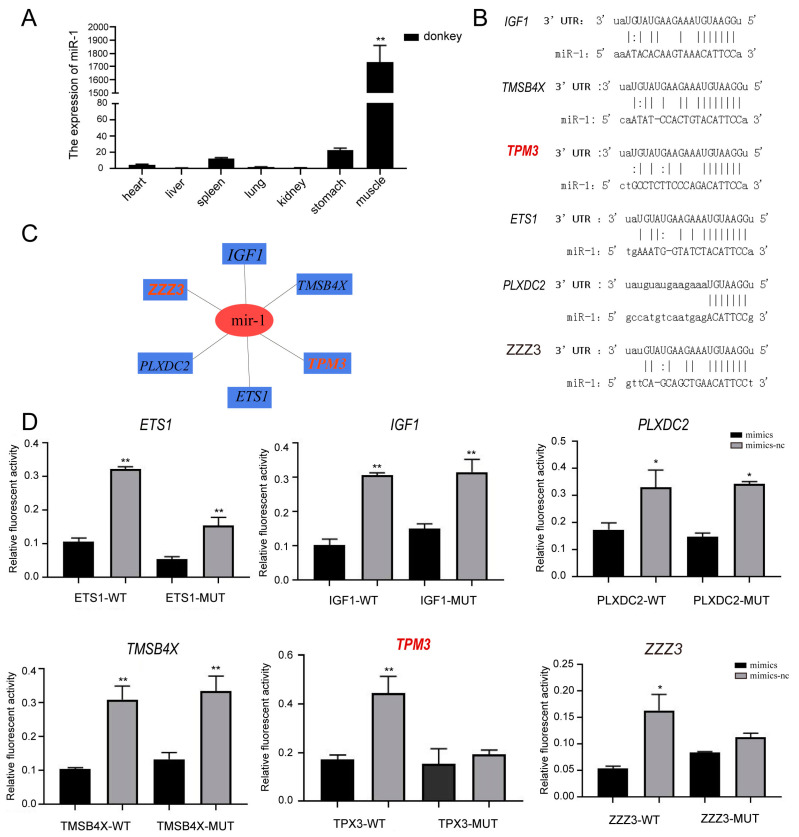
The eca-miR-1 target *EaTPM3*-5 in donkeys. (**A**) The expression profile among seven tissues in donkeys. (**B**) The network of eca-miR-1 and their target genes. (**C**) eca-miR-1 and the binding sites of its target gene. (**D**) The expression profile of the target genes in the WT samples and control group. * *p* < 0.05, ** *p* < 0.01.

Analyses were carried out to understand the protein-coding genes regulated by candidate miRNAs and their key roles in muscle development. Based on differentially expressed miRNAs and genes, we found that eca-miR-1 (sequence details: UGGAAUGUAAAGAAGUAUGUAU) showed different expression levels between S1 and S2 transcriptomes (Figure 3B; Table S1). The expression profiles of seven tissues showed that eca-miR-1 was highly expressed in muscle compared to the other six tissues (Figure 4A). Among these differentially expressed genes and miRNAs (Figure 3B), six genes were targeted by eca-miR-1 (Figure 4B,C). To further confirm the eca-miR-1 target genes, a dual-luciferase test was performed for eca-miR-1 and the predicted target genes that were transfected with the psiCheck2 vector (Figure 4C; Table S2). The relative expression profiles of V-Ets oncogene homolog 1 (*ETS1*), insulin-like growth factor-I (*IGF1*), Plexin domain-containing 2 (*PLXDC2*), and thymosin beta 4 X-linked (*TMSB4X*) exhibited a lower expression level in the wild-type samples compared to the normal control (NC), indicating that these genes may be target genes of eca-miR-1. We noticed that *TPM3* showed a significantly lower expression level in the wild-type samples than the NC, indicating that these two genes may be targeted by eca-miR-1 (Figure 4D). These results show that *TPM3* is a conserved target gene of eca-miR-1, which plays an important role in the muscle development of donkeys (Figure 4).

### 2.5. The TPM3 Gene Family Evolution and Expression Profile

**Figure 5:**
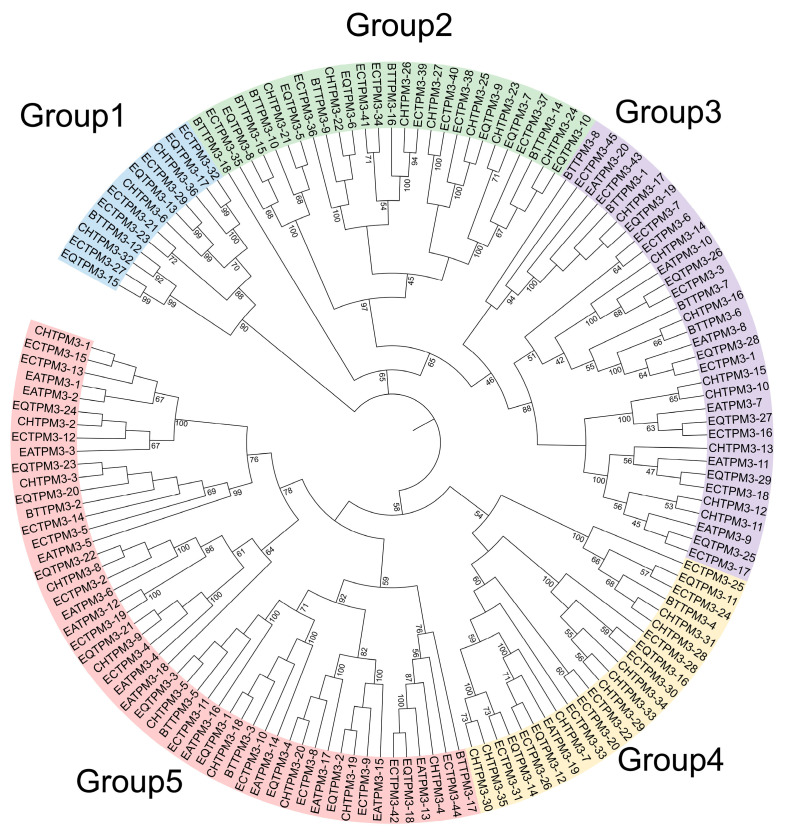
The phylogenetic tree of *TPM3* in horses, zebras, donkeys, cattle, and goats.

Based on the whole genome data of horses, zebras, donkeys, cattle, and goats, we characterized the gene family number of *TPM3* among the five genomes. We verified a total of 12 *TPM3* genes in zebras, 16 in horses, 17 in donkeys, 18 in cattle, and 20 in goats. Phylogenetic analysis of the *TPM3* gene family members of the five genomes showed that their genes were divided into five subgroups, and group 5 contained the greatest number of *TPM3* gene families (Figure 5; Table S3).

**Figure 6:**
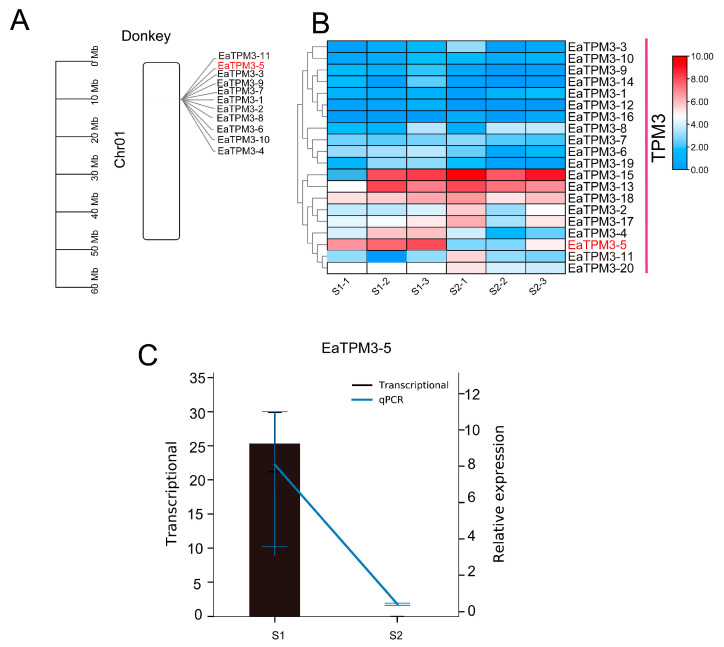
The expression profile of the muscle development-related gene family for *TPM3* in donkeys. (**A**) The chromosome location of the *TPM3* gene family in donkeys. (**B**) The expression profile of the *TPM3* gene family in donkeys. (**C**) The transcriptome profile and qPCR of *EaTPM3*-5. S1 indicates a 2-month-old Dezhou donkey; S2 indicates a 24-month-old Dezhou donkey. The bar plot is compared to the transcriptional analysis in the S1 and S2 stages on the left y-axis; the point plot is compared to the RT-PCR data in the S1 and S2 stages on the right y-axis. This is a double y-axis: the left y-axis represents the transcriptome expression level, and the right y-axis represents the RT-PCR level.

All *TPM3* genes of donkeys were located in chromosome 1 (Figure 6A). The transcriptome profiles of S1 and S2 showed that a total of seven *TPM3* genes exhibited a different expression profile, especially *EaTPM3*-5 (Figure 6B). The RT-PCR results showed the same pattern as the transcriptome data of S1 and S2 (Figure 6C; Table S4).

## 3. Discussion

### 3.1. Comparative Genomics and Collinearity Analysis Identify Muscle Development Gene TPM3 in Equus

The domestication of donkeys and horses has played an important role in human life, with these animals being valued for their draft ability, primarily based on their muscular strength [9,10]. Skeletal muscle plays an indispensable role in their bodies, as it is a dynamic tissue [37,38]. Both the contiguity and composition have been improved in the new horse genome. Donkey reference genome research is concerned with investigating the genetic basis of coat color and population genomics analyses. The quality of the zebra genome has also improved; however, the studies exploring these developments lack comparative studies of *Equus* genomes, especially their chromosome evolution [2,3,4,5]. Our study showed that the five species (donkeys, horses, zebras, cattle, and goats) contained 15,262 (47.43%) common genes, indicating that these species were evolutionarily conserved (Figure 1). With three high-quality reference genomes of *Equus*, a collinearity analysis was performed, and the results showed that nine chromosomes (Chr01, Chr02, Chr03, Chr06, Chr10, Chr18, Chr22, Chr27, Chr29, and Chr30) exhibited a good collinearity relationship, indicating that the genes in these chromosomes were evolutionarily conserved (Figure 2) [5,6]. In chromosome 1, a further microcolinearity analysis identified an evolutionarily conserved gene, *TPM3*, which is related to the function of muscle development (Figure 3) [7,8].

### 3.2. Comparative Transcriptomics Reveals the TPM3 Gene Potentially Involved in Muscle Development in Donkeys

Previous studies have shown that *TPM3* plays an important role in muscle development [15,16,17,18]. In order to further understand the function of *TPM3* involved in muscle development in equines, donkeys were selected as the representative animals for further analysis in this study. In this study, we used mRNA and miRNA sequencing to profile the skeletal muscle transcriptome and, thus, identify genes and miRNAs that were differentially expressed between donkeys with different feed efficiencies, including the transcriptome data of S1 (2-month-old Dezhou donkey) and S2 (24-month-old Dezhou donkey). The comparative transcriptome analysis results showed that a total of 45 miRNAs and 17 genes were differentially expressed, indicating that these miRNAs and genes may be related to muscle development in donkeys, which is consistent with previous studies [10,11,12,13,14]. Additionally, we found that eca-miR-1 was differentially expressed in S1 and S2; moreover, we also noticed that *TPM3* genes were differentially expressed in S1 and S2 transcriptomes (Figure 4). Interestingly, our collinearity analysis showed that *TPM3* was evolutionarily conserved in zebras, horses, and donkeys. Combining these two results showed that *TPM3* plays a critical role in muscle development, which is consistent with previous studies (Figure 4) [15,16,17,18].

### 3.3. TPM3 Regulates Muscle Development Targeted by eca-miR-1

Previous studies have reported that miR-21 is able to regulate arterial smooth muscle cell (ASMC) function by targeting tropomyosin 1 [39]. MiRNA-1 plays an important role in chordoma tissues [40] and various types of cardiac diseases [41]; miR-1, regulated by mammalian targeting of rapamycin (mTOR), has emerged as a key regulator of skeletal muscle development through governing the distinct stages of myogenesis [42], and the *SFRP1* gene is regulated by miR-1/206 and potentially affects skeletal muscle development [43]. In our study, eca-miR-1 was significantly highly expressed in muscle tissues compared to the other six tissues. We investigated the target gene of eca-miR-1, observing that the muscle development-associated gene *TPM3* was a target gene, as verified by the dual-luciferase tests transfected with the psiCheck2 vector, thereby supporting the role of eca-miR-1 in muscle development in donkeys (Figure 4). These results show that *TPM3* is a conserved target gene of eca-miR-1 in donkeys and plays an important role in muscle development, which is consistent with previous studies that showed that miRNA-1 can regulate the *TPM3* gene during muscle development (Figure 6) [39,40,41,42,43]. Based on these results, we specified the key members of the *TPM3* gene family in donkeys, and a total of 20 *TPM3* genes were identified (Figure 5). We observed that *EaTPM*-5 was significantly differentially expressed in the S1 and S2 stages in donkeys, as verified via RT-PCR (Figure 5). These results show that EaTPM-5, located in chromosome 1, plays a critical role in muscle development in donkeys (Figure 6) [15,16,17,18].

## 4. Materials and Methods

### 4.1. Ethics Statement

All experimental designs and procedures were performed according to the Regulations for the Administration of Affairs Concerning Experimental Animals (Ministry of Science and Technology, Xianyang, China, 2004). This study was approved by the Institutional Animal Care and Use Committee of Northwest A&F University (approval number: 20171208–010, 8 December 2017).

### 4.2. Comparative Genomics Analysis

Five species (*Equus quagga* (zebra), *Equus asinus* (donkey), *Equus caballus* (horse), *Bos taurus* (cattle), and *Capra hircus* (goat)) were selected for comparative genome analysis. OrthoFinder software version 2.4.0 with the default parameters was used to identify different types of orthologs [44].

### 4.3. Chromosome Collinearity Analysis Rearrangement Analysis

To detect the chromosome rearrangement events among *Equus caballus*, *Bos taurus*, *Equus quagga*, *Equus asinus*, *Capra hircus*, and *Equus ferus ssp. przewalskii*, pair-wise alignment of these chromosome-level genomes was performed using LAST software version 3.02. MCScan software version 1.1.11 was used to detect the synteny blocks and chromosome fusion events [45]. Mauve V2.4.0 with the default parameters was used for the visualization and detailed analysis of the aligned results [46].

### 4.4. Sample Collection

In this study, three male Dezhou donkeys at 2 months old (S1) and 24 months old (S2) were randomly selected as the test materials. After slaughter, the longissimus dorsi and biceps femoris muscles were taken, respectively, the size of each tube was the same, and the parts were as close to each other as possible. Then, the samples were immediately placed in liquid nitrogen and stored in a laboratory refrigerator at −80 °C for the subsequent extraction of total RNA (experimental animals were provided by Shandong Dong E E Jiao Co., Ltd., Shandong, China).

### 4.5. RNA Extraction and Sequencing Data Processing

RNA was extracted from the tissue using the Trizol method [45]. RNA concentration and quality were measured using a Nanodrop 2000, with 260/280 ratios ranging from 1.9 to 2.1. The quality of the original sequencing data was evaluated using FastQC version 0.10.1 [47], and the low-quality sequences in the original sequencing were removed to obtain high-quality sequences. HISAT2 software version 2.1.0 [47] was used to compare the clean reads with the reference genome (https://www.ncbi.nlm.nih.gov/genome/?term=equus+asinus, 15 December 2022), and the gene annotation files were downloaded to improve the alignment accuracy. The HISAT2 software version 2.1.0 strand-specific parameter was set to the following: --rna-strandness RF. The aligned reads were assembled using String Tie (v1.3.1) software [48]. The parameter settings were all the defaults. Each transcript was merged to obtain the complete sequencing information of each sample using Cuffmerge software version 2 [49]. Bowtie software version 0.12.5 [50] was used to align small RNAs with the reference genome to understand the distribution of small RNAs on the genome.

### 4.6. Identification of the miRNAs

The reads were aligned with known donkey miRNA precursor sequences, and the aligned reads were identified as known miRNAs. Then, based on the signature hairpin structure of the miRNA precursors, new miRNAs were predicted using miREvo software version 1.1 [51] and mirdeep2 software version 2.0 [52], and first base preference statistics were performed. The expression levels of all miRNAs (known miRNAs and new miRNAs) in the 6 samples were counted, and the expression levels were normalized via the TPM method. Based on the normalized results, DESeq2 [53] was used to quantitatively analyze the differential miRNAs with a standard *p* < 0.05.

### 4.7. Prediction and Validation of the miRNA Target Genes

To understand the biological functions of differentially expressed miRNAs, we used miRanda [54], PITA [55], and RNAhybrid [56] to perform target gene prediction analysis on the miRNAs. The miRNA–mRNA network interaction map was constructed via Cytoscape software version 3.6.1 [57]. The distribution of the candidate target genes in Gene Ontology was analyzed via GOseq analysis. In addition, KEGG analysis of the candidate target genes was performed via Pathway software version 23.0. Differential miRNAs were verified via RT-PCR. Quantitative primers were designed via Primer 5 and verified using NCBI Primer-BLAST to ensure accuracy. The primers of differentially expressed miRNAs were used for the RT-PCR (Table S1). Finally, they were synthesized via bioengineering, and U6 was used as the internal reference gene. At the same time, the miRNA–mRNA regulatory relationship was also quantitatively verified, and the quantitative results were calculated according to 2^−ΔΔct^ [58].

### 4.8. Vector Construction, Cell Transfection, and Dual-Luciferase Reporter Assay

The target fragment was cloned via PCR and then ligated to the psiCheck2 dummy digested with Xho I and Not I. 293T cells were cultivated in 96-well plates and divided into 6 groups for different treatments: the wild-type and mutant vector plasmids were co-transfected with eca-miR-1 mimics and mimics NCs, respectively. Fluorescence activity between different treatments was detected via the dual-luciferase assay. The primers are listed in Table S2.

### 4.9. Gene Family Identification Analysis

In this study, the identification of members of the *TPM3* gene family among the five species (*Equus quagga*, *Equus caballus*, *Equus asinus*, *Bos taurus*, and *Capra hircus*) was conducted using BLASTP software version 2.2.22 with an E-value of <1 × 10^−5^ and identity of ≥50% [59]. The candidate sequence protein domains were determined using PFAM [60], focusing only on proteins with *TPM3*.

To detect the *TPM3* members in *Equus asinus*, we downloaded a total of 20 *TPM3* members from NCBI and combined them with all *TPM3* genes in *Equus quagga*, *Equus caballus*, *Bos taurus*, and *Capra hircus* to construct a phylogenetic tree with MEGA [61]. We searched for the presence of potential domains of *TPM3* genes using the PFAM webserver [60]. The heatmap was visualized with TBtools [62]. The *TPM3* gene family member expression level was also verified via RT-PCR, and the primers are listed in Table S4.

### 4.10. Statistical Analysis

The test results were analyzed via one-way ANOVA using SPSS software version 20.0 [63]. * Indicates significant differences, *p* < 0.015; ** indicates extremely significant differences, *p* < 0.01.

## 5. Conclusions

This study provides the first analysis revealing chromosome-level evolution events and the evolutionarily conserved genes associated with muscle development, as well as their target miRNAs, in donkeys. Chromosome analysis showed that the three *Equus* species have undergone a significant number of chromosomal changes, but the skeletal muscle development-related gene, *TPM3*, has remained evolutionarily conserved among these three *Equus* species (donkeys, horses, and zebras), playing an important role in muscle development. Using the donkey as a model *Equus* species, a comparative transcriptome analysis was performed to investigate the function and network of *TPM3*, and the results showed that the *TPM3* gene was differentially expressed in S1 (2 months old) and S2 (24 months old) donkey muscles. Concurrently, eca-miR-1 was differentially expressed in S1 and S2 donkey muscles, indicating that it may play a role in muscle development. A dual-luciferase test further verified that the *TPM3* gene was targeted by eca-miR-1. In order to characterize the gene family member and the key member of the *TPM3* gene family, a total of 17 *TPMs* were identified in the donkey genome, and *EaTPM3-5* was significantly differentially expressed in S1 and S2 muscles, indicating that *EaTPM3-5*, located in donkey chromosome 1, is a key candidate gene involved in the muscle development of donkeys.

## Data Availability

All data generated or analyzed during this study are included in the published article.

## Supplementary Materials

The supporting information can be downloaded at https://www.mdpi.com/article/10.3390/ijms242015440/s1.
